# Efficacy and toxicity of Ipilimumab-Nivolumab combination therapy in elderly metastatic melanoma patients

**DOI:** 10.3389/fonc.2022.1020058

**Published:** 2022-11-07

**Authors:** Ronen Stoff, Shirly Grynberg, Nethanel Asher, Shachar Laks, Yael Steinberg, Jacob Schachter, Ronnie Shapira-Frommer, Guy Ben-Betzalel

**Affiliations:** ^1^ Ella Lemelbaum Institute of Immuno-Oncology, Sheba Medical Center, Ramat Gan, Israel; ^2^ Surgical Division, Sheba Medical Center, Ramat Gan, Israel

**Keywords:** melanoma, immunotherapy, geriatric oncology, anti PD-1, anti CTLA-4

## Abstract

**Introduction:**

Immunotherapy has revolutionized metastatic Melanoma therapy. The most active regimen is combination therapy of Ipilimumab-Nivolumab (Ipi-Nivo) with response rates (RR) of ~60% and median overall survival (OS) of ~6 years. Immune-related adverse events (irAE) are common (~60% develop grade 3-4) and pose a challenge when treating frail patients. We sought to examine whether Ipi-Nivo therapy is feasible in elderly metastatic melanoma patients.

**Methods:**

Electronic records of patients treated at the Ella Lemelbaum Institute with Ipi-Nivo between the years 2017-2021 were screened for age. Elderly patients were defined as age 75 and older (group A) and were matched with records of patients age <75 (group B). Records were analyzed for baseline parameters, immunotherapy regimen, RR, toxicity and progression-free survival (PFS).

**Results:**

Twenty-six relevant patients age >75 (median 77) were identified and were matched to 34 younger patients (median age 57). No statistically significant differences were noted in terms of baseline parameters except for BRAF mutation status (group A 15%, group B 47%, p=0.008). Response rate in group A was 38% and is consistent with previously published data. Median PFS was the same for both groups (A = 5.5 months, B= 7.5 months, p=NS). Treatment was similarly tolerated: 35% of group A patients completed 4 cycles of therapy compared to 28% for group B (p=NS). Grade 2-4 irAE were the same (A=58%, B=66%, p=NS) and there was no difference in the need for hospitalization for G3-4 events between the groups. (A=63%, B=69%, p=NS). Further division into 4 age groups (>80 vs 75-79 in group A and 65-74 vs <65 in group B) found no difference in terms of response rate or G3-4 toxicity.

**Conclusion:**

Ipilimumab-Nivolumab combination therapy in elderly metastatic Melanoma patients seems to be well tolerated and efficient in selected elderly patients based on performance status and comorbidities, just as in younger patients. This regimen seems to be a feasible treatment option for this age group.

## Introduction

Malignant melanoma is the 5^th^ most common cancer diagnosed in the US annually, yet only about 4% of the patients are diagnosed initially with a metastatic disease (1). Despite its relatively high prevalence in young adults, the majority (51.8%) of patients diagnosed are age 65 and over with about 25% of the patients being 75 and over. The median age at diagnosis is 65 (1).

For many years metastatic melanoma has been considered an aggressive and fatal malignancy for which no durable effective treatment was available. The overall median 1 and 5 year survival rate were 41% and 22% respectively as late as 2011, while for patients 75 and over the same 1 and 5 year survival rates were as low as 34% and 15% respectively (2).

The introduction of novel immunotherapy agents in the last decade has revolutionized the treatment in Melanoma with a marked increase in patients overall survival (2). The first agent to be introduced was Ipilimumab, an anti CTLA4 (Cytotoxic T-lymphocyte associated protein 4) agent which has shown improved survival as early as 2010 albeit with a significant toxicity profile and low response rates (3). The development of anti PD-1 (programmed death-1) agents such as Nivolumab and Pembrolizumab have further increased the efficacy with a much safer toxicity profile (4, 5).

The combination of Nivolumab and Ipilimumab was tested in the pivotal Checkmate 067 trial, and has given the best results to date with an improved response rate of 58% and a landmark 7.5 year survival rate of 48% (6, 7). Despite the significant improvement in both response rate and overall survival the combination therapy comes with an increased toxicity - 59% grade 3-4 immune related adverse events(irAE) and 31% of patients discontinuing treatment due to toxicity (6).

Due to its increased risk for toxicity many physicians opt not to use the combination in fragile patients and those with significant comorbidities or with pre-existing autoimmune disorders. Many physicians see elderly patients as fragile and therefore more prone to complications caused by possible immune related side effects. As about ¼ of the patients are 75 and over at diagnosis this poses a challenge when selecting the best treatment regimen for these patients.

The data regarding the use of single agent immunotherapy (anti PD-1 agents) in elderly patient are robust with most reports showing no difference in response rate, survival rate and toxicities when compared with younger patients (8–16). It is worth mentioning that in most studies the cut off age is 65 with only a small number of studies addressing the older population – 75 and over (17–20), though the results for this age group seem to be comparable if not superior to those of patients younger than 75. This seems to be the case mainly in Melanoma as a meta-analysis published by Nie et al. (21) has shown lower response rate for patients over 75 in comparison to younger patients with other types of solid tumors. The authors hypothesis for the difference between Melanoma and other tumor types is based on a study published by Samstein et al. in 2019 which showed that Melanoma patients over 75 have a higher tumor mutational burden (TMB) when compared to other tumor types of the same age group (22). Two other meta-analyses published has shown reduced survival for patients older than 75 across tumor types (23, 24). Many explanations have been suggested for the inferior response in elderly patients including age-related differences in T and B cell development (25), Macrophage polarization (26), T cell receptor (TCR) diversity (27) and intra-tumoral T regulatory (Treg) cell proportion (28).

As for the use of combination immunotherapy the prospective data is scarce with only 11% of patients in the combination arm of the Checkmate 067 trial being 75 or older (6). Data regarding the efficacy and toxicity in this subgroup was not published as the cut-off used in the subgroup analysis was 65. Prospective studies on the Ipilimumab-Nivolumab combination therapy in other tumor types (Renal cell carcinoma and Non-small cell lung cancer) showed that the combination regimen was not superior to the control arm for patients over 75, yet the dosing regimen for these indications is significantly different than Melanoma with an Ipilimumab dose of 1mg/kg every 6 weeks compared to the 3mg/kg every 3 weeks dose used in Melanoma (29, 30). Retrospective data about the combination is limited with one study from MSKCC that found only 8 patients 80 or over that were treated with the combination (31). Though the authors conclusion was that the rate of irAEs is similar to what was previously reported across all age groups, it is worth mentioning that out of the 8 patients reported 3 (37.5%) have developed immune mediated colitis requiring the use of Infliximab.

With Ipilimumab-Nivolumab combination treatment considered the most effective first line regimen for metastatic Melanoma patients, we sought in this study to evaluate the efficacy and toxicity of this regimen in patients aged 75 years and older.

## Methods

Electronic medical records of locally advanced unresectable or metastatic melanoma patients treated at the Ella Lemelbaum Institute were screened for age. Patients were included if they were treated with combination of Ipilimumab and Nivolumab between the years 2017 – 2021. Elderly patients were defined as age 75 and older (group A) and were matched with records of patients age <75 (group B). Records were analyzed for baseline parameters and response to therapy using a chi square test with a pre-defined alpha score of 0.05 for statistical significance. Progression-free survival (PFS) curves were assessed using the Kaplan-Meier method. Toxicity grading was done using the common terminology criteria for adverse events (CTCAE) v.5 (32). Given the relative frailty of elderly patients we chose to focus on all side effects with a special attention to side effects that were Grade 2 or higher. Toxicity comparison between the groups and subgroups (based on age and Melanoma subtype) was done using chi square test. All statistical analyses were done with Stata v.17.

Data was collected and analyzed in accordance with the local IRB approval.

## Results

Twenty-six relevant patients age >75 (median 77) were identified and were matched to 32 younger patients (median age 57). All patients in both groups received the standard dose regimen - Ipilimumab 3mg/kg and Nivolumab 1 mg/kg for an intended 4 cycles.

No statistically significant differences were noted between the groups in terms of baseline parameters except for BRAF mutation status (V600 Mutated patients - group A 15%, group B 47%, p=0.008). As for the Melanoma subtype there was a numerical difference between the groups with 30% of the older patients (group A) having either mucosal or uveal Melanoma while only 12% of the younger patients (group B) had these subtypes. However, the difference was not statistically significant.

Patient baseline characteristics are shown in **Table 1**.

**Table 1:** Baseline characteristics.

	Group A (over 75)	Group B (under 75)	
Median age (range)	77.5 (75–85)	57.5 (27-74)	
Melanoma subtype n (%) Cutaneous Acral Mucosal Uveal Unknown	14 (54%) 2 (8%) 5 (19%) 3 (11%) 2 (8%)	24 (75%) 2 (6%) 2 (6%) 2 (6%) 2 (6%)	p=NS
BRAF V600 Mutation n (%) Mutated Wildtype Unknown	4 (15%) 21 (81%) 1 (4%)	15 (47%) 17 (53%) 0 (0%)	p=0.008
ECOG PS n (%) 0 1 2 3 Unknown	14 (54%) 10 (38%) 1 (4%) 1 (4%) 0 (0%)	20 (62%) 6 (19%) 4 (12%) 0 (0%) 2 (6%)	p=NS
Serum LDH n (%) Normal range Elevated <X2 UNL Elevate >X2 UNL Unknown	16 (61%) 8 (31%) 1 (4%) 1 (4%)	20 (62%) 4 (12%) 4 (12%) 4 (12%)	p=NS
Disease stage AJCC-8 n (%) Inoperable stage III M1a M1b M1c M1d	2 (8%) 2 (8%) 1 (4%) 14 (53%) 7 (27%)	1 (3%) 5 (16%) 2 (6%) 10 (31%) 14 (44%)	p=NS
Line of therapy n (%) 1 2 3	17 (65%) 7 (27%) 2 (8%)	19 (59%) 11 (34%) 2 (6%)	p=NS

ECOG PS, eastern cooperative oncological group performance status; LDH, lactate dehydrogenase; AJCC, American joint committee on cancer; NS, nonsignificant.

nonsignificant

Response evaluation was based on iRECIST (immunotherapy response and evaluation criteria in solid tumors) (33) using either computer tomography (CT) or Positron-Emission Tomography - Computer Tomography (PET-CT) along with clinical evaluation of visual and palpable skin lesions where applicable.

Response rate was numerically, but not significantly, higher in the younger patients’ group (group A = 38%, group B = 56%, chi^2^ test, p=NS). The response rate in group B was similar to what has been previously reported while the response in group A was lower [with the exception that 30% of group A were either mucosal or uveal Melanoma, both known to have a lower response rate in comparison to cutaneous Melanoma (34, 35)]. The rate of Partial Response (PR) was about the same for both groups (23% v 25%) with younger patients achieving more Complete Responses (CR) (31% vs. 15%).

When examining only the cutaneous Melanoma patients in both groups (14 in group A and 24 in group B) the response rate was 29% in group A with 2 patients achieving PR (14%) and 2 achieving CR (14%), whereas in group B the response rate was 67% with 6 patients achieving PR (25%) and the other 42% achieving CR. This difference was statistically significant with a p-value of 0.0232. On the other hand, non-cutaneous primaries had a 50% response rate in group A and 25% in group B (p=NS).

Response patterns are detailed in **Tables S1–S3** in the appendix, yet a direct comparison between the groups is problematic due to different histological subtypes in each group.

**Figure 1:**
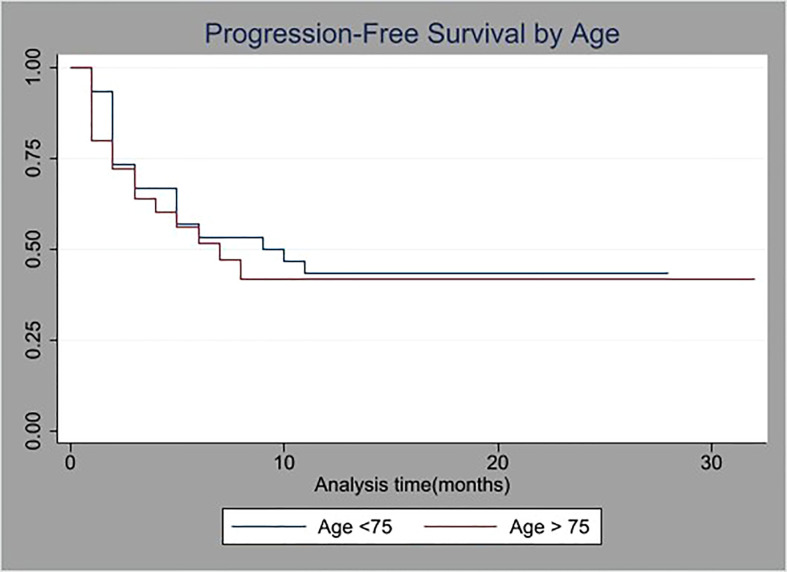
Kaplan-Meier survival curves of progression-free survival (PFS) stratified by age (cutoff at 75 years old).

The median progression free survival (PFS) was 5.5 months for group A and 7.5 months for group B (p=NS). Kaplan-Meier curve for PFS is illustrated in **Figure 1**.

With regard for toxicity – the treatment was similarly tolerated: 35% of the older patients (group A) completed 4 cycles of therapy compared to 28% of the younger patients (group B) (p=NS). Median number of cycles was 3 for both groups. Treatment was stopped early due to toxicity for 12 patients in group A (46%) and 18 patients in group B (56%), (p=NS). Grade 2-4 irAEs requiring treatment with glucocorticosteroids were noted in 15 patients in group A (58%) and 21 patients in group B (66%) (p=NS).

Grade 3-4 adverse events were noted in 11 patients in group A (42%) and 16 patients in group B (50%). The most common side effects were colitis (7 patients in each group) and hepatitis (3 patients in group A and 9 patients in group B). It is worth mentioning that though there were 7 patients in group A who developed colitis, only 4 of them developed G2-4 Colitis and none required the use of Infliximab. In contrast, out of the 7 G3-4 colitis patients in group B, 3 (43%) required the use of Infliximab.

Overall, 7 patients were hospitalized in group A (63%) and 11 patients were hospitalized in group B (69%, p=NS). The reasons for hospitalization in group A were hepatitis in 2 patients and one of each – pneumonitis, colitis, dermatitis, neurological toxicity (polyradiculopathy) and gastritis. In the long term follow up all toxicities were resolved except for the patient with pneumonitis who required chronic steroid treatment. In group B the most common reason for hospitalization was hepatitis in 6 patients with 2 more developing colitis and the others being 1 case each of pneumonitis, myositis and hypophysitis. All these toxicities in group B were resolved, except for the patient with hypophysitis who required long-term hormonal replacement with Prednisolone and Levothyroxine.

For patients who developed G3-4 toxicity treatment rechallenge with single agent Anti-PD-1 was done for 3 patients in group A with a median time to rechallenge of 2 months (1–4), whereas in group B 10 patients were rechallenged with a median time of 2.5 months (1.5-12). There were no documented flairs of toxicities during the rechallenge in both groups. The common side effects are summarized in **Table 2**.

**Table 2:** Immune related adverse events.

IrAEs	Group A- n (%)	Group B - n (%)	
Any grade	20 (77%)	22 (69%)	p=NS
G2-4	15 (58%)	21 (66%)	p=NS
G3-4	11 (42%)	16 (50%)	p=NS
Use of GCs	15 (58%)	21 (66%)	p=NS
Hospitalization	7 (27%)	11 (34%)	p=NS
Colitis Any grade G3-4 Use of infliximab	7 (27%) 2 (8%) 0/7 (0%)	7 (22%) 4 (25%) 3/7 (43%)	p=NS
Hepatitis G3-4	3 (11%)	7 (22%)	p=NS
Misc. Hypophysitis Rash Neurological Myositis	1 (4%) 3 (11%) 1 (4%) 0 (0%)	2 (6%) 0 (0%) 0 (0%) 2 (6%)	

irAEs, immune related adverse events; GCs, glucocorticosteroids; G, grade; NS, not significant.

Since most previous reports used 65 as the cut-off for elderly patients an analysis of subgroups based on age was done with both groups being divided into two subgroups. in group A we assessed patients aged 75-79 versus those over 80 and in group B we assessed those aged 65-74 versus those less than 65. There was no statistically significant difference in terms of response rate or G3-4 toxicity between the 2 subgroups in both group A and group B. Results of toxicity according to age subgroups are shown in **Table 3**, results for response rates are available at **Table S4** in the appendix.

**Table 3:** – Subgroup analysis of toxicity.

Subgroup age (n)	G3-4 Toxicity n (%)	
>80 (8)	3 (37.5%)	p=NS
75-79 (18)	8 (44%)	reference
65-74 (12)	8 (67%)	p=NS
<65 (20)	8 (40%)	reference

G, grade; NS, nonsignificant.

## Discussion

The rapid incorporation of immunotherapy for malignant Melanoma has changed the oncological outcomes drastically, albeit at the cost of significant immune related toxicities. The majority of the patients diagnosed are 65 years or older. The use of single agent anti-PD1 for this age group has been reported in multiple studies with no significant differences in efficacy or toxicity when compared to younger patients.

The use of the immunotherapy combination blockade of anti-PD1 and anti CTLA-4 (Ipilimumab+Nivolumab) has shown the best results in metastatic Melanoma patients to date, but at the cost of about 60% G3-4 immune related adverse events. Consequently, most elderly Melanoma patients are considered by medical personnel to be unfit for the combination therapy due to concerns regarding these patients’ ability to endure the mentioned toxicities. Of special interest is the group of patients that are over 75 who represent about 25% of all newly diagnosed Melanoma patients. Unlike the robust data regarding the use of anti-PD1 single agent therapy in this age group there is only scarce data available about the use of the combination therapy. We collected data about the use of the combination therapy in this age group in our institution and compared it to a cohort of patients younger than 75. The 26 elderly patients identified represent about 15-20% of all patients over 75 treated in our institution at the same time span, while the majority of the rest were treated with a single agent, and a small minority were treated with best supportive care only. The overall results are encouraging both in terms of efficacy and mainly toxicity, as currently the main concern for many physicians is the adverse events profile of the combination therapy representing a difficult clinical dilemma. Our study did not find any statistically significant difference in the toxicity profile – treatment duration was similar between both groups as they underwent the same number of treatment cycles (median=3 for both). Both groups of patients also developed a similar rate of G2-4 events requiring treatment with systemic glucocorticosteroids (58% v 66%). Moreover, numerically there were more G3-4 toxicities for the younger patients (50% v 42%). The rate of patients requiring hospitalization was similar (27% v 34%). Younger patients also required more often the use of glucocorticosteroids or more advanced immunosuppression (Infliximab) while none of the older patients required use of Infliximab. When examining the colitis patients (7 in each group) it is worth noting that none of the 14 patients had primary mucosal Melanoma and most of the patients had cutaneous Melanoma (5 in group A, 6 in group B) with the rest being uveal Melanoma (2 in group A, 1 in group B) and 1 patient with an unknown primary. These toxicity results are very encouraging and are different than those previously published by the MSKCC group (31) in which 37% of patients over 80 required Infliximab for immune mediated colitis following combination immunotherapy. The reason for such a difference is unclear and could be explained by the fact that doctors are more prone to using advanced immune modulation for elderly patients in fear of complications and lengthy hospitalizations in this relatively frail age group. A biological basis for this phenomenon needs further elucidating.

Rechallenge was attempted in 3 of 11 patients with G3-4 toxicity in group A (27%) and in 10 of 16 patients in group B (62%). This difference is hard to interpret due to the small number of patients in each group but might be attributed to the fact that the majority of those in group B had Hepatitis for which rechallenge is accepted in cases where steroid treatment is effective, and the tapering is successful.

In terms of response there was a numerical difference in the response rate between the two groups in favor of the younger patients, which can be attributed to the difference in the Melanoma subtypes. About 30% (vs. 12% in the younger cohort) of the elderly patients were treated for either mucosal or uveal melanomas, both known to be much less responsive to immunotherapy compared with cutaneous Melanoma (34, 35). This relatively high proportion of non-cutaneous Melanoma patients in the elderly group is probably because since these subtypes are less responsive to immunotherapy, the treating physicians were more likely to prefer combination therapy for these patients. These differences are accentuated when looking into the cutaneous Melanoma subgroup, which showed a much higher response rate for the younger patients (67% v 29%) which was statistically significant. An interesting result was for the non-cutaneous Melanoma patients in group A which showed a 50% response rate versus a 25% response rate for the same patients in group B. this difference was not statistically significant due to the small number of patients in each group (12 in group A and 8 in group B).

When further dividing the groups into age subgroups we found no significant differences in terms of response rate or Grade 3-4 toxicity between those aged 75-79 and those aged more than 80 (in group A) and between those aged 65-74 and those aged <65 (in group B).

Progression-Free Survival was statistically similar for both groups as is shown in **Figure 1**.

## Conclusions

To our knowledge this is the largest reported series of elderly patients treated with combination immunotherapy, and we believe it carries a significant impact.

Ageism is a common difficult issue in modern oncology therapy stemming probably from physicians` tendency to overestimate the general difficulty in treating older patients. The encouraging results shown in this study in efficacy and more importantly toxicity in these elderly patients has led us the conclusion that a patient’s age shouldn’t be a contraindication for the use of immunotherapy combination. A case-by-case approach is warranted when deciding on the treatment regimen, incorporating age, performance status, comorbidities, and the status of the cancer into consideration.

This study does have a few limitations though. As a retrospective analysis it presents inherent biases that are difficult to overcome, the main one being the unequal distribution of the different Melanoma subtypes between the 2 groups with more cutaneous Melanoma patients in the younger patient group. This difference interferes with our ability to infer proper conclusions when assessing the response rate, yet it doesn’t affect the toxicity profile which was comparable for both groups. Furthermore, as these patients were treated on a clinic bases some of them did not fit RECIST criteria for evaluation and were evaluated clinically. Lastly, although this is the largest series of its kind it is still limited in number of patients thus requiring caution in making definitive conclusions. Further prospective studies are warranted for validation, for elucidating the mechanisms of response and for developing predictive factors for toxicity in these patients.

## Data availability statement

The datasets presented in this article are not readily available because by local MOH law datasets are not to be exported out of the country. Requests to access the datasets should be directed to Ronen Stoff ronen.stoff@mail.huji.ac.il.

## Ethics statement

The studies involving human participants were reviewed and approved by Sheba medical center IRB - approval SMC 4387-17. Written informed consent for participation was not required for this study in accordance with the national legislation and the institutional requirements.

## Author contributions

RS: • Data analysis • Statistical planning • Manuscript preparation • Draft revision. SG: • Data analysis • Statistical analysis • Manuscript preparation • Draft revision. NA: • Database management • Manuscript preparation. SL: • Manuscript preparation • Draft revision. YS: • Manuscript preparation • Draft revision. JS: • Manuscript preparation • Draft revision. RS-F: • Statistical analysis • Draft revision. GB-B: • Manuscript conceptualization • Data analysis • Statistical analysis • Manuscript preparation. All authors contributed to the article and approved the submitted version.

## Acknowledgments

The authors would like to thank Sheba Medical center for its continuing support, the management of the Ella institute led by Prof Schachter and Dr. Shapira. A special appreciation goes to all our patients and their caregivers.

## Conflict of interest

The authors declare that the research was conducted in the absence of any commercial or financial relationships that could be construed as a potential conflict of interest.

## Publisher’s note

All claims expressed in this article are solely those of the authors and do not necessarily represent those of their affiliated organizations, or those of the publisher, the editors and the reviewers. Any product that may be evaluated in this article, or claim that may be made by its manufacturer, is not guaranteed or endorsed by the publisher.
