# Supplementary material for: Efficacy and toxicity of Ipilimumab-Nivolumab combination therapy in elderly metastatic melanoma patients
Source: Front Oncol. 2022 Nov 7;12:1020058. doi: 10.3389/fonc.2022.1020058 (PMC9676931; doi:10.3389/fonc.2022.1020058)
Supplement: Supplementary file 1 [file DataSheet_1.pdf]

Appendix – response rates

Table S1 - Response patterns in all patients

Response n (%)	Group A (n=26)	Group B (n=32)
ORR	10 (38%)	18 (56%)
PR	6 (23%)	8 (25%)
CR	4 (15%)	10 (31%)
SD	3 (12%)	1 (3%)
PD	13 (50%)	13 (41%)

Abbreviations: ORR – overall response rate; PR – partial response; CR – complete response; SD – stable disease; PD – progression of disease

Table S2 - Response patterns in cutaneous Melanoma

Response n (%)	Group A (n=14)	Group B (n=24)
ORR	4 (29%)	16 (67%)
PR	2 (14%)	6 (25%)
CR	2 (14%)	10 (42%)
SD	2 (14%)	0 (0%)
PD	8 (57%)	8 (33%)

Abbreviations: ORR – overall response rate; PR – partial response; CR – complete response; SD – stable disease; PD – progression of disease

Table S3 - Response patterns in non-cutaneous Melanoma

Response n (%)	Group A (n=12)	Group B (n=8)
ORR	6 (50%)	2 (25%)
PR	4 (33%)	2 (25%)
CR	2 (17%)	0 (0%)
SD	1 (8%)	1 (12.5%)
PD	5 (42%)	5 (62.5%)

Abbreviations: ORR – overall response rate; PR – partial response; CR – complete response; SD – stable disease; PD – progression of disease

Table S4 - Response rate according to age subgroups

Subgroup age (n)	Response rate n (%)	
>80 (8)	3 (37.5%)	p=NS
75-79 (18)	7 (39%)	reference
65-74 (12)	7 (58%)	p=NS
<65 (20)	11 (55%)	reference

Abbreviations: NS=nonsignificant
